# Etiologies and hearing status in bilateral vestibulopathy: a retrospective study of 315 patients

**DOI:** 10.3389/fneur.2023.1271012

**Published:** 2023-11-29

**Authors:** Julie Moyaert, Bieke Dobbels, Olivier Peetermans, Bram Boon, Florence Lucieer, Nils Guinand, Griet Mertens, Annick Gilles, Paul van de Heyning, Angelica Pérez Fornos, Raymond van de Berg, Vincent Van Rompaey

**Affiliations:** ^1^Department of Translational Neurosciences, Faculty of Medicine and Health Sciences, University of Antwerp, Antwerp, Belgium; ^2^Department of Otorhinolaryngology and Head and Neck Surgery, Antwerp University Hospital, Edegem, Belgium; ^3^Division of Balance Disorders, Department of Otorhinolaryngology and Head and Neck Surgery, Faculty of Health Medicine and Life Sciences, School for Mental Health and Neuroscience, Maastricht University Medical Center, Maastricht, Netherlands; ^4^Service of Otorhinolaryngology Head and Neck Surgery, Department of Clinical Neurosciences, Geneva University Hospitals, Geneva, Switzerland; ^5^Faculty of Physics, Tomsk State Research University, Tomsk, Russia

**Keywords:** bilateral vestibulopathy, hearing loss, COCH protein, human, causality, Meniere disease

## Abstract

**Importance:**

The development of a vestibular implant has reached milestones and seems to be a promising therapeutic tool for bilateral vestibulopathy (BV). Given the former lack of therapeutic options for BV, the disease has received scant attention in the previous research literature. It is therefore of major importance to gain more insight into the underlying pathology of BV. Furthermore, as some research groups specifically use a combined vestibulo-cochlear implant, the size of the group of BV patients with associated hearing loss is of special interest.

**Objectives:**

The study aimed to determine the definite and probable etiology in bilateral vestibulopathy (BV) patients and to report on their hearing status.

**Design:**

This study involves multicenter retrospective study design.

**Setting:**

The research setting is at tertiary referral centers.

**Participants:**

Consecutive BV patients diagnosed at the Antwerp University Hospital between 2004 and 2018 at the Maastricht University Medical Center between 2002 and 2015 and at the Geneva University Hospital between 2013 and 2018, who met the BV diagnostic criteria of the Bárány Society.

**Main outcome measures:**

Primary interests were the etiology and hearing status of BV patients. Moreover, the data of vestibular tests were examined (caloric irrigation, rotatory chair tests, and video-head impulse test).

**Results:**

The authors identified 315 BV patients, of whom 56% were male patients. Mean age at diagnosis was 58.6 ± 15.1 (range 7–91) years. The definite cause was determined in 37% of the patients and the probable cause in 26% of the patients. No cause was identified in 37% of BV patients. The largest subgroup included patients with genetic etiology (31%), most frequently COCH mutation. Only 21% of patients (*n* = 61) had bilateral normal hearing. Almost half of the patients (45%, *n* = 134) had profound hearing loss in at least one ear.

**Conclusion:**

BV is a heterogeneous condition, with over a third of cases remaining idiopathic, and nearly three-quarters affected by hearing loss. COCH mutation is the most common non-idiopathic cause of BV in our population. Only 21% of our BV patients presented with bilateral normal hearing.

## Highlights

– Question: What is the underlying etiology and the hearing status of patients with bilateral vestibulopathy (BV)?
– Findings: In this three-center cohort-study that included 315 patients, the most frequent non-idiopathic cause of BV is genetic (31%). A proportion as high as 79% of BV patients suffers from uni- or bilateral-associated hearing loss.
– Meaning: BV is a heterogenous disease. The majority of BV patients have an associated hearing loss. This justifies the development of a combined vestibulo-cochlear implant. Furthermore, this implicates that the current research investigating if vestibular loss contributes to cognitive decline should correct for the possible associated hearing loss of study subjects.

## Introduction

Bilateral vestibulopathy (BV) is characterized by a bilateral partial or complete loss of function of vestibular structures of the inner ear, vestibular nerves, or a combination of both (1). BV is a rare condition with a prevalence of 28 per 100,000 US adults (2). However, this might be an underestimation as this prevalence was solely based on questionnaires in the US National Health Survey. Oscillopsia and gait imbalance are the classic complaints of BV patients. However, recent research suggests that BV patients might suffer from a broader spectrum of symptoms, including spatial disorientation and anxiety (3, 4).

Given the former lack of therapeutic options for BV, the disease has received scant attention in the previous research literature. However, several major advancements in the field have currently drawn attention to BV. First, different groups have recently reached milestones in the development of a vestibular implant (5, 6). Inspired by the concept of the cochlear implant, the vestibular implant aims to transmit motion information of a patient’s head to the brain via electrical currents that stimulate the vestibular nerve branches directly. Furthermore, to restore both vestibular and auditory function, a combined vestibular (VI) and cochlear implant (CI) device, also known as the vestibulocochlear implant (VCI) was developed. This prototype is currently being investigated by the Maastricht University Medical Center (UMC) and the University of Geneva. In addition, countries such as Spain, Italy, and Belgium are cooperating in the development of the bionic vestibular implant for bilateral vestibulopathy (BionicVest project) (7).

Despite the encouraging results in the development of the vestibular implant, many challenges remain. Therefore, it is essential to gain more insight into the pathophysiology of BV. Additionally, the prevalence of severe hearing loss in BV patients might be interesting for the future development of vestibular devices (8).

Second, both animal and human research have suggested a link between the vestibular system and cognition (4). Due to the loss of the peripheral vestibular input in BV patients, these patients are a preferred test population group to unravel the negative cognitive repercussions of vestibular loss. However, most of the previous studies did not correct the hearing status of the enrolled patients (4). As hearing loss is an established risk factor for cognitive decline and dementia (9, 10), this might be an overlooked factor. To be able to estimate the importance of hearing loss when evaluating the link between cognition and vestibular loss, it is necessary to know the prevalence of hearing loss in BV patients. Indeed, if BV patients frequently suffer from an associated hearing loss, their previously observed cognitive deficits might be (at least partially) due to their hearing loss instead of solely their vestibular loss.

Lastly, regarding the close anatomical relationship between the cochlea and the vestibular organ, hearing loss and vestibular loss seem to be intertwined. Previous studies have revealed the prevalence of vestibular hypofunction of approximately 50% in a population with sensorineural hearing loss (11, 12). In this study, the prevalence of hearing deficits will be assessed in a population with severe vestibular hypofunction. This might enable a better understanding of the relationship between hearing loss and vestibular loss.

This study seeks to obtain data on the main clinical features of 315 BV patients, which is, to date, the largest group of BV patients reported in the literature. Our aim is to gain insight into the broad spectrum of etiologies causing BV as this might be instrumental in the current development of new therapeutic strategies such as the vestibular implant. Furthermore, this study assesses the prevalence of hearing loss in BV patients which is currently of particular interest to further unravel the relationship between cognition, hearing loss, and vestibular loss.

## Materials and methods

### Ethical considerations

This study was performed in accordance with the recommendations of the Ethics Committee of the Antwerp University Hospital (UZA), which approved the protocol (number 16/42/426). For patients diagnosed at the Maastricht University Medical Center (MUMC) and at the Geneva University Hospital, ethical approval was not required in accordance with the guidelines outlined by the medical research involving human subjects act.

### Study design and setting

A retrospective descriptive multi-center study was performed on consecutive BV patients diagnosed at the ENT departments of UZA (between 2004 and 2018), Maastricht UMC+ (between 2002 and 2015), and GUH (between 2013 and 2018).

### Participants

In accordance with the diagnostic criteria for BV, which have been established by the Bárány Society (1), patients with bilaterally reduced vestibular response were included, in cases as follows:

1. caloric response: sum of bithermal maximum peak slow phase velocity on each side <6°/s
2. rotatory chair: horizontal angular vestibulo-ocular reflex gain <0.1 upon sinusoidal stimulation
3. video-head impulse test (vHIT): bilaterally pathological horizontal angular vestibulo-ocular reflex gain <0.6.

### Outcome measures

#### Etiologies

Underlying etiologies were classified into different groups and subgroups. Moreover, a classification of certainty into definite or probable etiology was adopted similar to previous studies (13, 14). A definite diagnosis of BV was determined when the patient displayed abnormal results on both bedside and laboratory examinations alongside symptoms. On the other hand, a probable diagnosis was made when either the bedside or laboratory results were unusual, combined with symptoms.

#### Hearing status

Pure-tone averages (PTA) were at frequencies of 1,000, 2,000, and 4,000 Hz.

### Measurement

#### Vestibular tests

Standardized testing of vestibular function was necessary for the inclusion of BV patients. Bilateral bithermal caloric irrigations were performed with water at 30 and 44°C under standard conditions. Rotatory chair tests were performed using sinusoidal rotation at 0.05 Hz with a peak velocity of 60°/s for the patients at the AUH and GUH and at 0.1 Hz with a peak velocity of 100°/s for patients at the MUMC. These vestibular tests were recorded using electronystagmography in the AUH and MUMC and using videonystagmography in GUH. The horizontal vHIT was performed using the commercially available vHIT goggles (Otometrics in AUH and MUMC, Synapsis System in GUH).

#### Demographics

Age at diagnosis and gender were obtained by reviewing clinical records. The time of diagnosis was based on the date when the vestibular tests were performed.

#### Etiology

All qualitative and quantitative data required for diagnosis were obtained by reviewing clinical records. This included medical history, family history, vestibular tests, and non-vestibular tests such as neuro-ophthalmologic examination, laboratory tests, and cerebral imaging to diagnose the underlying cause. These non-vestibular tests were only performed when relevant (i.e., when the underlying etiology was uncertain). If multiple hearing tests were available, the one closest to the time of diagnosis was chosen. The diagnosis of Menière’s disease, cerebellar ataxia with neuropathy and bilateral vestibular areflexia syndrome (CANVAS), and Cogan’s syndrome were made in accordance with the international diagnostic criteria (15–17).

#### Hearing status

The hearing assessment consisted of pure tone audiometry, performed in a sound-proof room. The average of the air-conduction thresholds at the frequencies 1,000, 2000, and 4,000 Hertz, in the high Fletcher index (F.i.), was calculated for each ear. Afterward, each ear was categorized into five grades of hearing impairment in accordance with the classification of hearing loss by the World Health Organization:

(1) no hearing loss (F.i. ≤ 25 dB HL), (2) mild hearing loss (25 dB HL < F.i. ≤ 40 dB HL), (3) moderate hearing loss (40 dB HL < F.i. ≤ 60 dB HL), (4) severe hearing loss (60 dB HL < F.i. ≤ 80 dB HL), and (5) profound hearing loss (F.i. > 80 dB HL). Furthermore, patients were classified as having symmetrical hearing when results of both ears were classified in the same category of hearing impairment. When both ears were classified in a different category of hearing impairment, the patients were classified as having asymmetrical hearing. If multiple hearing tests were available, the one closest to the time of diagnosis was chosen.

### Data collection

The obtained data were coded and collected in each center. Due to patient’s privacy, the database is not added in Appendix. All analyses were carried out with SPSS 25 in UZA.

## Results

### Participants

In total, 315 patients with a confirmed BV diagnosis according to the Bárány Society criteria were included in the study. Of these patients, 129 were diagnosed at UZA, 137 at MUMC, and 49 at GUH.

### Vestibular tests

Only patients who met the BV criteria established by the Bárány Society were included in the study. A bilaterally reduced caloric response was found in 207 (67%) patients. A reduced gain on rotatory chair testing was seen in 217 (72%) of BV patients and in 55 (85%) results of the vHIT were impaired. However, since the vHIT test was not routinely used in UZA and MUMC until recently, only 65 patients underwent vHIT testing. Caloric testing and rotatory chair testing were not performed in 9 and 15 included patients, respectively.

### Demographics

The mean age at diagnosis was 58.6 ± 15.1 (range 7–91) years. Of the study population, 56% were male patients and 44% were female patients.

### Etiologies

**Figure 1:**
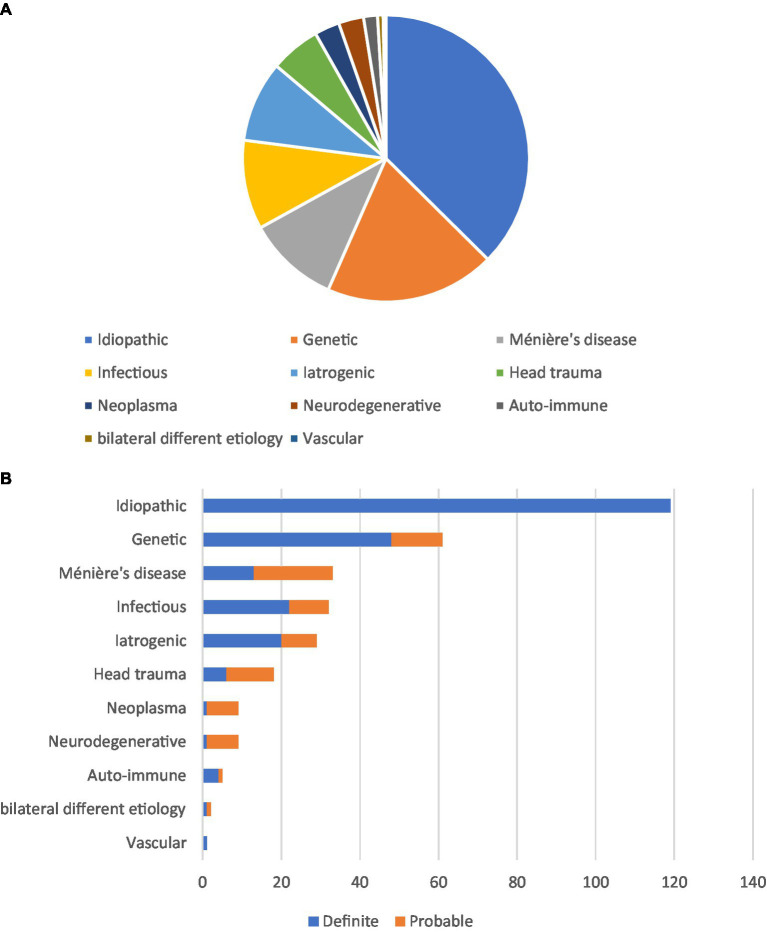
Etiologies in patients with bilateral vestibulopathy. **(A)** Etiologies in 315 patients with BV, and no cause could be identified in 119 patients. The most frequent non-idiopathic cause was genetic. **(B)** A classification of certainty into definite or probable etiology was adopted for all etiologies.

Overall, the definite etiology of BV was determined in 37% of patients and the probable etiology in 26% of patients. In 37%, the etiology of BV remained idiopathic. The most frequent non-idiopathic etiologies included genetic disorders (31%), Menière’s disease (17%), infectious diseases (16%), iatrogenic complications (15%), and head trauma (9%). Etiologies are presented in Figure 1. The different subgroups will now be discussed.

#### Genetic disorders

Of all patients, 61 were diagnosed with a genetic disorder. A definite diagnosis with a genetic confirmation COCH mutation was most prevalent (*n* = 46). In two patients, a definite genetic etiology was found due to DNMT1 and CHD7 mutations. In 13 patients, clinical presentation and family history were highly suggestive of hereditary etiology although genetic examinations were either undiagnostic or not performed. In addition, seven patients were diagnosed with suspected CANVAS.

#### Menière’s disease

BV was caused by Menière’s disease and/or Menière’s disease treatment in 33 patients. Only the 13 patients with bilateral Menière’s disease were classified as having definite etiology causing BV. The remaining 20 patients suffered from unilateral Menière’s disease, without cochlear symptoms, such as hearing loss, on the unaffected ear. As the vestibular deficit on the unaffected side could not be explained by a definite diagnosis of Menière’s disease according to the criteria, these patients were classified as BV with a probable etiology of Menière’s disease. Due to the incapacitating Menière’s disease vertigo spells, eight patients (four of whom bilateral and four unilateral Menière’s disease) had already been treated with vestibular neurectomies or intratympanic gentamicin (18). In one patient with unilateral Menière’s disease, a labyrinthectomy was performed. In these patients, it could not be determined to what extent Menière’s disease itself was responsible for the BV.

#### Iatrogenic complications

Iatrogenic complications were the causative factor of BV in 29 patients. It should be highlighted that treatments of Menière’s disease and vestibular schwannomas were not included in this group. In 20 patients, BV developed after intravenous antibiotic treatment, namely, gentamicin (*n* = 17), tobramycin (*n* = 1), streptomycin (*n* = 1), and neomycin (*n* = 1). One of these patients was regarded as having a probable etiology since gentamicin administration was only mentioned in history but was not stated in the clinical records. In one patient, BV probably developed as a complication of bilateral cochlear implantation. Furthermore, BV probably developed after heart or liver surgery in two patients, where it was presumably caused by the administration of antibiotics. In two patients, chemotherapy was the definite cause of BV as symptoms developed closely after administration (one oxaliplatin and one unknown oncologic treatment found place in another hospital). Finally, in four patients, tuberculostatic drugs, radiotherapy for laryngopharyngeal cancer (*n* = 2), and quinine (antimalarial drug) were the probable causative factor of BV.

#### Infectious diseases

Regarding the infectious causes (*n* = 31), BV was definitely caused by meningitis in 19 patients, one of whom developed viral meningitis as a complication of unilateral labyrinthitis. Meningitis was regarded as a probable cause in one patient; while meningitis was not reported on history taking, imaging modalities were highly suggestive of meningitis as the underlying etiology. Two cases definitely resulted from bilateral neuritis vestibularis. Furthermore, one patient was diagnosed with unilateral vestibular neuritis and one with unilateral labyrinthitis and were both classified as probable since the vestibular deficit on the “unaffected” side could not be explained. Lyme’s disease was the probable cause of BV in two patients. BV was probably caused by Epstein–Barr viral infection in one patient and by the mumps in another patient (given positive serology). The last three cases most likely resulted from a congenital infection (rubella, *n* = 1 and cytomegalovirus, *n* = 2).

#### Head trauma

In the group of head trauma (*n* = 18), a bilateral skull base fracture caused a definite BV in six patients. In the other 12 patients, the association with BV was most likely related to their head trauma since their BV symptoms developed after the incident. These 12 patients presented with mild head trauma without the need for hospitalization (*n* = 3), severe traumatic brain injury with the need for hospitalization (*n* = 1), unilateral skull base fracture (*n* = 6), and whiplash injury (*n* = 2).

#### Others

In one patient, the vestibular loss was thought to be related to extreme alcohol abuse. In another patient, a metabolic etiology was suspected since this patient suffered from diabetes mellitus. All these etiologies were classified as probable. Furthermore, seven patients were diagnosed with unilateral neoplasm, which was most likely the cause of their BV. Of these patients, five had a unilateral vestibular schwannoma, one had a right-sided cerebellar hemangioma, and one had a skull base meningioma located in the right cavernous sinus. One patient with neurofibromatosis had undergone bilateral resection of a vestibular schwannoma. In five patients, BV was caused by an autoimmune disease. Among them, Cogan’s syndrome was regarded as the definite cause in four patients and Sjögren’s syndrome was the probable cause in one patient. One patient developed BV after a cerebrovascular accident in the brainstem and was therefore regarded as a definite vascular cause. Finally, in two patients, a different etiology was identified on each inner ear (meningitis and head trauma; cochlear implantation and labyrinthitis).

#### Idiopathic

No cause could be identified in 118 patients.

### Hearing status

Of the 315 patients, the pure tone audiometry of 297 patients was obtained, and in 18 patients, no data were available. In most patients (61%, *n* = 180), symmetric hearing was observed. Only 21% of patients (*n* = 61) had bilateral normal hearing. Almost half of the patients (45%, *n* = 134) had profound hearing loss in at least one ear. Exploring the hearing status of patients with different etiologies, the most prominent finding was that as hearing deteriorated, the proportion of genetic cause increased. The results of hearing performance are shown in Table 1. The hearing function of patients was categorized into two groups: the best ear and the other ear. Patients with symmetric hearing function are displayed on the diagonal of the table. Furthermore, a comparison of the group’s hearing status with age-related hearing loss reveals that over 75% have poorer hearing than what we would predict based on their age. Additionally, since the cause of BV is unknown in 37% of cases, it would be worthwhile to examine the hearing status of this cohort. The analysis reveals that 17% exhibit normal hearing, 17% experience mild hearing loss, 11% display moderate hearing impairment, 14% suffer from severe hearing loss, and 40% have profound hearing loss.

**Table 1:** Results of hearing performance.

Best ear	Other ear				
	Normal	Mild	Moderate	Severe	Profound
Normal	**61**	12	8	4	6
Mild		**21**	18	5	9
Moderate			**8**	15	18
Severe				**11**	22
Profound					**79**

Bold values mean the number of patients with symmetrical hearing.

## Discussion

### Etiology

The most frequent etiologies included idiopathic (37%), genetic disorders (31%), Menière’s disease (17%), infectious diseases (16%), and iatrogenic complications (15%).

Genetic etiology is the most frequent non-idiopathic cause in our BV population, with a prevalence of 31%. This is strikingly high compared with previous study cohorts, where none of the BV patients was suspected to have a genetic etiology (14, 19–21). Nearly all our patients with genetic etiology were affected by a COCH mutation, causing DFNA9 disease. This is the most common type of autosomal dominant non-syndromic deafness in Belgium and the Netherlands (22). In the patients recruited at GUH, none were suspected to have genetic etiology. The high prevalence of DFNA9 in the Netherlands and Belgium might thus be a likely explanation for the high proportion of genetic causes in this study. Hence, caution should be taken to extrapolate these results to other BV populations.

In our study population, no cause could be identified in 37% of patients. This is comparable to previous literature, where the prevalence of idiopathic BVP ranges from 21 to 51% (3, 14, 19–21). Furthermore, in our study, the definite etiology of BV was determined in 37% and the probable etiology in 26%, which is roughly consistent with previous studies (13, 14). However, the distinction between definite and probable etiology is often artificial and subjective.

It should be highlighted that all BV patients were recruited from an ear-nose-throat (ENT) department. BV patients with accompanied hearing problems (Menière’s disease, vestibular schwannoma, DFNA9, etc.,) might be overrepresented in this cohort compared to studies including patients from a neurological clinic. In the latter, BV patients with neurodegenerative causes will be more prevalent than in our cohort.

In several patients, their underlying etiology could not fully explain the BV and was therefore classified as probable etiology. In the twelve probable head trauma patients, it is possible that BV was caused by an accompanying labyrinthine concussion and/or traction injury of the vestibulocochlear nerves although this was never objectified (23). A probable explanation for the 19 unilateral Menière’s disease patients is that the vestibular function can already be impaired bilaterally, while cochlear symptoms are only apparent on one side. A previous study noted abnormal saccular function in 27% of the “unaffected” ears in unilateral Menière’s disease (24). Moreover, endolymphatic hydrops was present in the unaffected ear in more than 55% of patients with unilateral Menière’s disease (25). These observations have important implications for vestibular destructive therapy in intractable unilateral Menière’s disease, e.g., intratympanic gentamycine or neurectomy. In this case, it would be essential to perform vestibular testing before applying destructive therapy for unilateral Menière’s disease to avoid including bilateral vestibulopathy.

In the eight patients with a unilateral neoplasm and in patients with unilateral neuronitis, a predisposed, idiopathic vestibular loss contralaterally might have been present prior to manifestation of the culprit neoplasm or infection. Furthermore, for some etiologies such as neurodegenerative and metabolic diseases, it is more difficult to prove a definite association.

### Hearing status

In 79% of our patients, unilateral or bilateral hearing loss was observed. Bilateral profound hearing loss was found in 27% of all BV patients and 19% of BV patients suffered from unilateral profound hearing loss. Compared with the literature, the prevalence of hearing loss is substantially higher in our BV cohort (see Table 2).

**Table 2:** Hearing loss observed in other BV cohorts reported in the literature.

Study	*N* (mean age ± SD in years)	Inclusion criteria	Hearing status
Lucieer, 2016	154 (60 + − 12.5)	Imbalance and/or oscillopsia and summated SPV < 20°s bithermal caloric tests	Mean PTA _(1000, 2000, and 4,000 Hz)_ < 40 dB: *N* = 86, 56% Mean PTA _(1000, 2000, and 4000 Hz)_ 40–85 dB: *N* = 41, 27% Mean PTA _(1000, 2000, and 4000 Hz)_ > 85 dB: *N* = 16, 10% Missing data: *N* = 11, 7%
Zingler, 2009	255 (62 + − 16)	Pathological HIT and/or SPV <5°/s on each caloric irrigation	Normal hearing: *N* = 179, 69%
Rinne, 1998	53	Absent caloric and/or rotational response	Mean PTA _(1000, 2000, and 4000 Hz)_ < 40 dB: *N* = 26, 49% Mean PTA _(1000, 2000, and 4000 Hz)_ 40–85 dB: *N* = 19, 36% Mean PTA _(1000, 2000, and 4000 Hz)_ > 85 dB: at least *N* = 2, 4%
Vibert, 1995	52 (aged from 3 to 82 years)	Complete loss of response to both caloric and rotatory pendular testing	Normal hearing idiopathic group: *N* = 10, at least 19% No data non-idiopathic group
Sun, 2014	15 (65 + − 10)	Not specified	Normal hearing: *N* = 9, 60% Presbyacusis: *N* = 4, 27% Missing data: *N* = 2, 13%

Demographic characteristics, inclusion criteria, and hearing status of bilateral vestibulopathy patients in previous studies.

HIT, head impulse test; SPV, slow peak velocity; PTA, pure tone average; SD, standard deviation.

Since most of the included DFNA9 patients suffered from a bilaterally profound hearing loss, the high prevalence of DFNA9 patients in this BV cohort is likely to be the reason for the high occurrence of hearing loss in our BV cohort compared to previously described BV cohorts. Another explanation for the high proportion of BV patients with hearing loss might be the ENT setting in this study compared to a neurological setting in other studies. Most of these BV patients will visit primarily an ENT clinic because of their hearing loss instead of their vestibular loss.

### The link between hearing loss, vestibular loss, and cognition

BV seems frequently associated with hearing loss. Previous studies have pointed out a high prevalence of BV in patients with hearing loss (12).

This is of particular interest for the previous research demonstrating a link between vestibular loss and cognitive decline in BV patients. Hearing loss is a well-known risk factor for cognitive decline (10). Therefore, the observed cognitive deficits in BV patients could be due to their hearing loss instead of their vestibular loss. However, most of the previous studies did not correct the hearing status of the enrolled BV patients (4). Given the rising incidence of dementia, it is important to accurately identify risk factors for cognitive decline. Future research evaluating if vestibular loss could be associated with cognitive impairment should correct for hearing loss. Vice versa, research investigating cognition in a hearing-impaired population should correct for vestibular function. However, recent research evaluates the association between cognition, hearing, and vestibular loss in BV patients. This study indicates that hearing loss is correlated with poorer scores on both the immediate memory and language subscales, as well as the total RBANS-H scale. Conversely, vestibular loss was found to be associated with reduced performance on the attention subscale of the RBANS-H (26).

### Vestibular implants and rehabilitation prospects

Vestibular implants are promising research devices that are being investigated to rehabilitate BV patients. Several fundamental milestones have been reached in the development of the vestibular implant (5), including the restoration of the vestibulo-ocular and vestibulo-spinal reflexes (27, 28). Moreover, besides the laboratory improvement, the vestibular implant also seems to lead to a functional benefit for BV patients. Less complaints of oscillopsia can be expected as the dynamic visual acuity in BV patients went from highly impaired to close to normal scores by switching the implant device (29).

As hearing loss is still an important issue in vestibular implantation, the Geneva–Maastricht team developed a combined vestibulo-cochlear implant, and all implanted ears in their studies were deaf. These patients therefore benefitted from useful hearing rehabilitation in everyday life, while vestibular electrodes were only activated in a laboratory setting. More accurate knowledge of the hearing status in BV patients as well as potential vestibular implantation with hearing preservation is crucial to assess the need for a pure vestibular implant and the necessity of a hybrid vestibulo-cochlear implant (30). Note that the present study shows that a proportion as high as 46% of patients have at least unilateral profound hearing loss.

Many of the BV patients in this study have a COCH mutation causing DFNA9 disease. DFNA9 is characterized by mutated cochlin deposits in the inner ear that lead to strangulation and degeneration of the dendritic fibers. Moreover, DFNA9 is known to be accompanied by severe degeneration of the cochlear sensory axons and dendrites (31). Given the dendritic damage in DFNA9 patients, a poorer outcome of cochlear or vestibular implantation might be hypothesized due to aberrant stimulation of the affected nerve fibers. Four DFNA9 patients have been instrumented with a vestibulo-cochlear implant by the Geneva–Maastricht group with suboptimal laboratory results (32). Even though the sample size is small, it can be hypothesized that this subgroup of patients might therefore not be ideal candidates for vestibular implantation, at least in the experimental phase. However, previous research demonstrated DFNA9 patients to benefit equally from a cochlear implant as a comparative control group (31).

## Limitations

Given the retrospective nature of this study, we were reliant on the integrity of the medical record data. The data in these medical records were gathered for the purpose of clinical consistency and not with the aim of providing information for any specific study. A more directed approach to a prospective study would solve some problems encountered, such as incomplete availability of laboratory results or family history and might have resulted in a decreased proportion of patients with an idiopathic etiology.

Since the underlying etiology of BV is often unclear, the interpretation of medical records by experienced researchers plays a key role in determining the final underlying etiology. In this study, participants were recruited at three different medical centers, and thus, medical records were not reviewed by the same group of researchers. However, all centers are well-recognized and experienced referral centers in the field of vestibulology. Moreover, vestibular tests were not performed under the same standardized conditions but rather depended on the preferred standardization of the respective institution.

## Conclusion

BV is a heterogeneous condition, with over a third of cases remaining idiopathic, and nearly three-quarters affected by hearing loss. COCH mutation is the most common cause of BV in our population, and the hearing status of these patients is substantially worse than that of patients with other etiologies.

## Data availability statement

The raw data supporting the conclusions of this article will be made available by the authors, without undue reservation.

## Ethics statement

The studies involving humans were approved by the Ethics Committee of the Antwerp University Hospital (UZA). The studies were conducted in accordance with the local legislation and institutional requirements. The participants provided their written informed consent to participate in this study.

## Author contributions

JM: Writing – original draft, Writing – review & editing. BD: Writing – original draft, Writing – review & editing. OP: Writing – review & editing. BB: Writing – review & editing. FL: Writing – review & editing. NG: Writing – review & editing. GM: Writing – review & editing. AG: Writing – review & editing. PH: Writing – review & editing. AF: Writing – review & editing. RB: Writing – review & editing. VR: Writing – review & editing.
